# Tirzepatide and Cardiovascular Outcomes: A Narrative Review of Mechanisms, Efficacy and Implications for Heart Failure Management

**DOI:** 10.1002/edm2.70152

**Published:** 2026-01-22

**Authors:** Hamza A. Abdul‐Hafez, Ameer Awashra, Sosana Bdir, Sarah Saife, Qasem Salah, Mohammed Barbarawi, Thabet Swaileh, Ahmed Emara, Mohamed S. Elgendy, Abdalhakim Shubietah

**Affiliations:** ^1^ Department of Medicine An Najah National University Nablus Palestine; ^2^ Department of Internal Medicine, Faculty of Medicine Al‐Azhar University Cairo Egypt; ^3^ Faculty of Medicine Tanta University Tanta Gharbia Egypt; ^4^ Department of Medicine Advocate Illinois Masonic Medical Center Chicago Illinois USA

**Keywords:** cardiovascular outcomes, heart failure, management, review, tirzepatide

## Abstract

**Background:**

Tirzepatide, a dual GIP/GLP‐1 receptor agonist, offers a novel cardiometabolic strategy beyond glycemic control with important implications for heart failure care. By producing potent, sustained weight reduction and favourable changes in lipids, blood pressure, systemic inflammation and endothelial biology, tirzepatide targets central pathophysiologic drivers of obesity‐related HFpEF.

**Methods:**

We conducted this review to synthesise current evidence on the mechanisms, clinical efficacy and therapeutic implications of tirzepatide for heart failure management, with emphasis on obesity‐related HFpEF, cardiorenal effects and safety considerations. Randomised clinical programmes and the SUMMIT outcomes trial have demonstrated symptomatic and functional improvements, reverse cardiac remodelling on imaging, reduced circulating markers of myocardial stress and fewer worsening heart‐failure events versus placebo, alongside signals of renal stabilisation.

**Results:**

The tolerability profile aligns with the GLP‐1 class, with gastrointestinal events predominating and a low risk of clinically important hypoglycemia; biliary events may be more likely at higher doses, while pancreatitis risk has not been clearly elevated. Data in HFrEF remain limited and caution is advised given prior mixed results with incretin therapies and theoretical concerns about rapid weight loss in advanced systolic failure.

**Conclusion:**

This review integrates mechanistic insights and contemporary trial evidence to clarify how dual incretin agonism may modify the trajectory of obesity‐driven heart failure, to inform multidisciplinary clinical decision making, and to highlight key unanswered questions and research priorities needed to define tirzepatide's full role in heart failure management.

## Graphical abstract

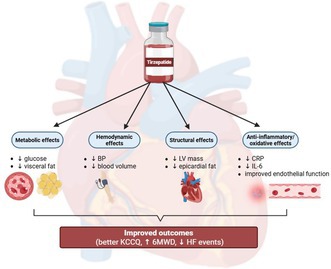

**Abbreviations**
ACE: angiotensin‐converting enzyme
ARB: angiotensin receptor blocker
ASCVD: atherosclerotic cardiovascular disease
BP: blood pressure
CKD: chronic kidney disease
CMR: cardiac magnetic resonance
CRP: C‐reactive protein
CV: cardiovascular
DBP: diastolic blood pressure
EF: ejection fraction
eGFR: estimated glomerular filtration rate
FMD: flow‐mediated dilation
GIP: glucose‐dependent insulinotropic polypeptide
GLP‐1: glucagon‐like peptide‐1
GLP‐1RA: GLP‐1 receptor agonist
HbA1c: haemoglobin A1c
HF: heart failure
HFmrEF: heart failure with mildly reduced ejection fraction
HFpEF: heart failure with preserved ejection fraction
HFrEF: heart failure with reduced ejection fraction
IL‐6: interleukin‐6
LV: left ventricle/left ventricular
LVEF: left ventricular ejection fraction
MACE: major adverse cardiovascular events
NT‐proBNP: N‐terminal pro‐B‐type natriuretic peptide
NYHA: New York Heart Association
PGIS: patient global impression of severity
PWV: pulse‐wave velocity
SBP: systolic blood pressure
SGLT2: sodium‐glucose cotransporter 2
T2DM: type 2 diabetes mellitus

## 1 Introduction

Heart failure (HF) is one of the biggest causes of mortality, morbidity, frequent hospitalizations and poor quality of life worldwide [1, 2]. An estimated 64 million individuals worldwide are impacted; it is anticipated that as the population ages and a higher incidence of cardiovascular risk factors such as hypertension, diabetes and obesity occurs, the prevalence of HF would rise [1, 3]. Most recent projections for the US suggest an increase in the prevalence of HF by about 46% from 2012 to 2030, with a corresponding increase in healthcare expenses by roughly 127% [1]. Recent years have witnessed substantial advancements in the diagnosis and treatment of this critical condition. Several international HF associations jointly released a universal definition and classification standard for HF. HF is defined as ‘a clinical syndrome with symptoms and/or signs caused by a structural and/or functional cardiac abnormality and corroborated by elevated natriuretic peptide levels and/or objective evidence of pulmonary or systemic congestion’ [4]. The classification of HF according to ejection fraction (EF) has been updated to include HF with reduced ejection fraction (HFrEF; EF ≤ 40%), HF with mildly reduced ejection fraction (HFmrEF; EF 41%–49%), and HF with preserved ejection fraction (HFpEF; EF ≥ 50%) [4].

Pharmacological therapy, such as angiotensin‐converting enzyme (ACE) inhibitors, beta‐blockers, angiotensin receptor blockers (ARBs) and diuretics, has traditionally been the primary method to controlling HF [5, 6]. The primary objective of these medications is to relieve symptoms, reduce fluid retention and improve heart function [7]. Currently, there is evidence from recent clinical trials that tirzepatide, an incretin mimetic, has proven beneficial in HF management [8, 9]. The phase 2 trials of tirzepatide produced promising results, prompting the beginning of the SURPASS programme. It includes multiple phase 3 randomised controlled trials to evaluate the efficacy and safety of tirzepatide in patients with type 2 diabetes mellitus (T2DM). The SURPASS programme contains a total of 10 phase 3 trials. Five of these trials have been finished and published [10].

Tirzepatide is a promising medication that activates both the glucose‐dependent insulinotropic polypeptide (GIP) and glucagon‐like peptide 1 (GLP‐1) receptors, revolutionising the treatment of T2DM as an adjunct to diet and exercise [11, 12]. Tirzepatide has been demonstrated to improve glycemic control by lowering glycosylated haemoglobin and improving fasting and postprandial glucose levels when compared to other diabetic medications [11]. Furthermore, the studies show a reduction in body weight and other cardiovascular advantages by altering the lipid profile, lowering blood pressure and visceral adiposity levels [8]. Tirzepatide enhanced functional ability, quality of life and reduced the risk of worsening HF events in obese patients with HFpEF, with benefits observed even among those with chronic kidney disease, according to the SUMMIT trial. Along with improved kidney function and patient outcomes, these effects could help to further support cardiovascular health [8]. Tirzepatide was also tested in the SURPASS‐CVOT trial on patients with T2DM and atherosclerotic cardiovascular disease, where it was expected to give clear evidence of cardiovascular safety and superiority over dulaglutide, a GLP‐1 receptor agonist, in terms of lowering significant adverse cardiovascular events [9].

Despite these significant treatment advancements, HF continues to present significant challenges. Management of medical conditions can be further complicated by adverse effects, medication intolerances, non‐adherence to complex drug regimens and failure to address the underlying processes of HF [7]. Early intervention and ongoing treatment are crucial to optimising long‐term outcomes. This narrative review seeks to contribute to the existing knowledge on tirzepatide for HF management by comprehensively analysing the mechanisms of action beyond glycemic control, the clinical efficacy and outcomes and main implications for HF management and tolerability profile.

## 2 Tirzepatide: Molecular Actions and Physiologic Pathways

Tirzepatide is a dual GIP/GLP‐1 receptor agonist with high potency at GIPR and substantial GLP‐1R activity. At the pancreatic islet level, tirzepatide potentiates the incretin effect: GLP‐1R activation markedly increases β‐cell cAMP and glucose‐dependent insulin secretion while indirectly suppressing α‐cell glucagon (via intra‐islet somatostatin) during hyperglycemia [13]. Concurrent GIPR stimulation further amplifies insulin release via PI3K–AKT signalling and, in preclinical models, supports β‐cell proliferation and survival [14]. In vitro, tirzepatide induces supra‐additive β‐cell cAMP and insulin secretion compared with GLP‐1 or GIP alone [13, 14]. The net result is improved postprandial glucose control and diminished hepatic gluconeogenesis. These islet effects alleviate chronic hyperglycemia and its metabolic stress on the heart and vasculature, providing a rationale for downstream cardiometabolic benefits.

Tirzepatide's extra‐pancreatic actions also contribute importantly to its cardiometabolic profile. In the central nervous system, GLP‐1R and GIPR signalling converge on hypothalamic feeding circuits to increase satiety and reduce appetite [13]. GLP‐1 in particular is a well‐characterised anorectic signal, and animal studies indicate that GIPR activity in the brain also promotes satiety. These central effects translate into reduced caloric intake and weight loss, alleviating obesity‐related cardiac loading. In adipose tissue, GIP receptors are highly expressed; GIP signalling upregulates lipoprotein lipase and triglyceride uptake, promoting efficient storage of dietary lipids in metabolically healthy adipocytes [14]. By contrast, GLP‐1 has limited direct receptor expression in adipocytes, and its effects are largely indirect via autonomic and metabolic pathways, including increased sympathetic tone driving lipolysis of stored triglycerides [13]. Together, these complementary actions improve adipocyte function. Tirzepatide increases insulin sensitivity, adiponectin secretion and the lipid‐buffering capacity of subcutaneous fat, thereby reducing ectopic lipid deposition in muscle, liver and myocardium and ameliorating systemic insulin resistance [13, 14].

In the cardiovascular system and kidneys, tirzepatide's GLP‐1–GIP signalling may exert both direct and indirect protective effects. GLP‐1 receptors are distributed widely (brain, pancreas, kidney, vasculature) [15]. In humans, cardiac GLP‐1R expression appears enriched in the sinoatrial node and conduction tissue rather than in working ventricular myocardium [16, 17]. Although the importance of direct cardiac GLP‐1R signalling remains under study, incretin agonists improve myocardial metabolism and cell survival (reducing apoptosis and hypertrophy) and enhance endothelial function [15, 17]. GLP‐1 receptor agonists (GLP‐1RAs) also promote vascular vasodilation via cAMP/PKA and nitric oxide pathways and reduce inflammation (lowering reactive oxygen species and pro‐inflammatory cytokines), effects that mitigate atherosclerosis and hypertension.

In the kidney, GLP‐1R activation inhibits the sodium–hydrogen exchanger NHE3 in proximal tubules, leading to natriuresis and diuresis [18]. GLP‐1–induced release of atrial natriuretic peptide (ANP) and suppression of renal angiotensin II further augment salt excretion, lowering intravascular volume and blood pressure. Chronic GLP‐1RA therapy also reduces glomerular hyperfiltration, oxidative stress and profibrotic signalling in the kidney. Together, these renal effects unload the failing heart by attenuating volume overload and blood pressure, while reduced adiposity and systemic inflammation further relieve myocardial workload. Importantly, sustained weight loss and improved glycemic control decrease cardiac wall stress, improve lipid profiles, and lessen insulin resistance, mechanisms that are thought to underlie the favourable cardiovascular outcomes observed with incretin‐based therapies [14, 15, 17].

## 3 Clinical Evidence of Tirzepatide: Cardiovascular, Cardiorenal and Cardiometabolic Outcomes

**FIGURE 1:**
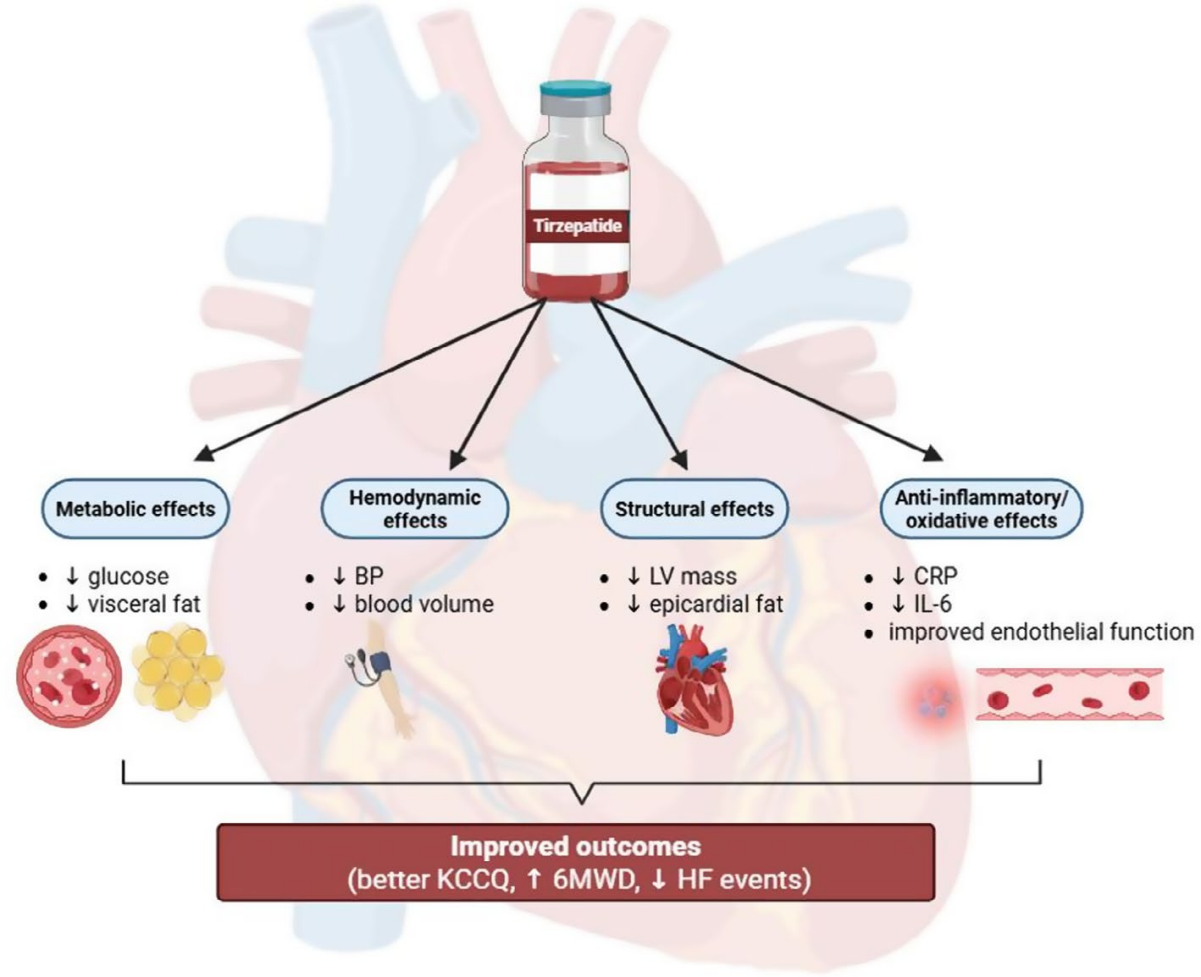
Integrated framework for tirzepatide in HFpEF management. Through metabolic, haemodynamic, structural and anti‐inflammatory pathways, tirzepatide modifies the disease trajectory of HFpEF, leading to improvements in symptoms, functional capacity and reduction in HF events.

**FIGURE 2:**
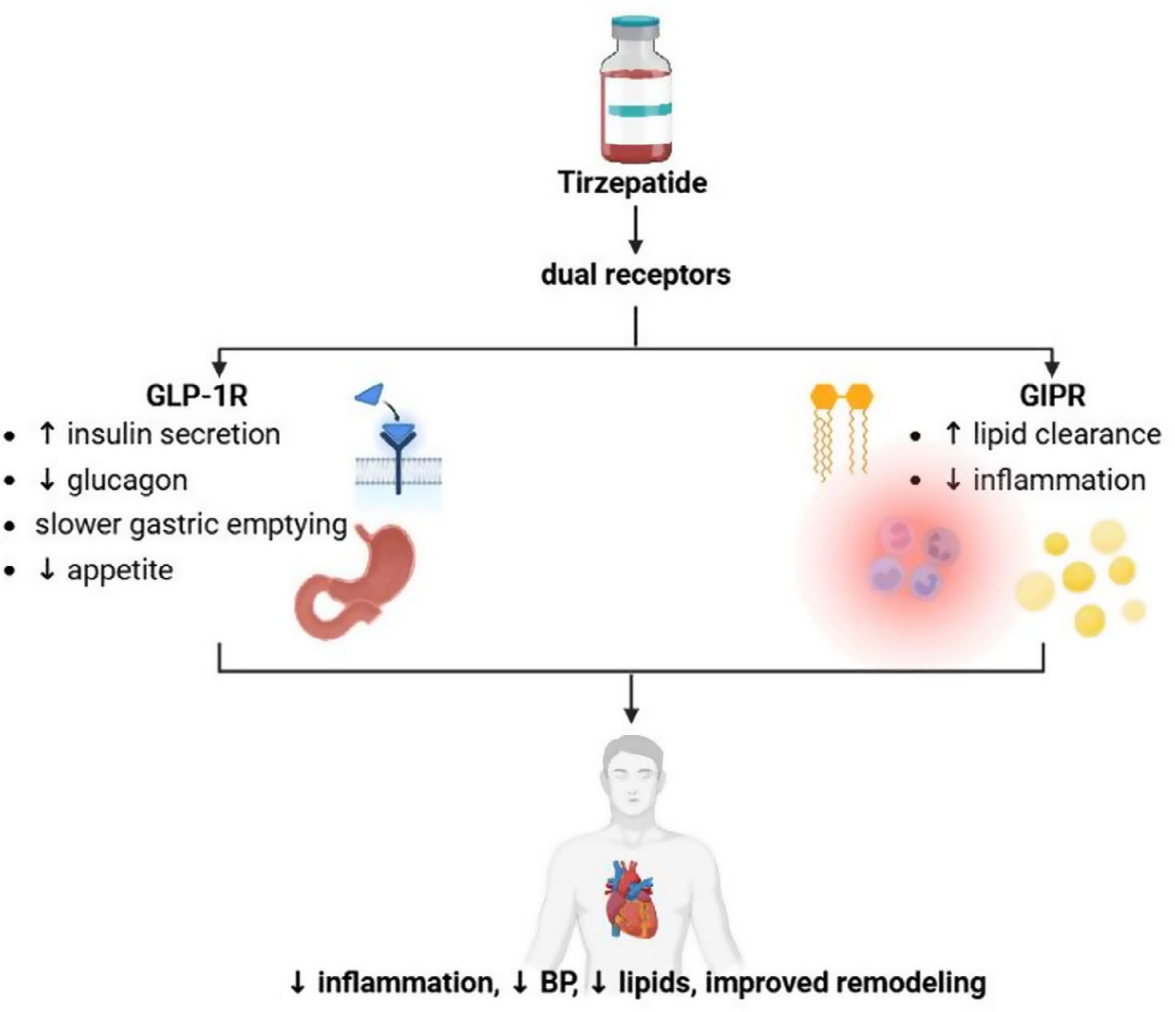
Mechanistic pathways of tirzepatide beyond glycemic control. By activating both GLP‐1 and GIP receptors, tirzepatide enhances insulin secretion, improves lipid metabolism, reduces systemic inflammation and lowers blood pressure. These effects translate into cardiovascular benefits, including reduced cardiac remodelling, improved vascular compliance and enhanced functional capacity.

Tirzepatide has demonstrated significant benefits across several cardiometabolic risk factors, including hypertension, obesity, dyslipidemia and diabetes. Although its role in preventing major cardiovascular events remains under investigation in ongoing large‐scale outcome trials, consistent improvements in metabolic parameters, body composition and inflammation suggest potential cardiovascular protective effects (Figures 1 and 2).

### 3.1 Tirzepatide in Blood Pressure Regulation

In SURPASS and related trials, once‐weekly tirzepatide (5–15 mg) produces modest, consistent BP reductions: systolic by ~4–6 mmHg and diastolic by ~2 mmHg over ~1 year [19]. SBP reduction was primarily weight‐loss–mediated. In SURPASS‐4, weight‐independent effects explained 33%–57% of the difference in SBP [20, 21].

A clear dose–response is seen in SURPASS‐J monotherapy, where higher doses yielded larger on‐treatment reductions (≈11/5–6 mmHg at the highest dose). In pooled SURPASS analyses, those starting with SBP > 140 mmHg had the largest reductions, whereas participants with low‐normal baseline SBP (< 122 mmHg) showed minimal change, indicating a downward shift in BP most pronounced among hypertensive patients [21, 22].

In the obesity (non‐diabetic) trial (SURMOUNT‐1), tirzepatide 72‐week treatment led to clinically significant BP improvements at the patient level. Notably, 58.0% of tirzepatide‐treated participants achieved normal BP (< 130/80 mmHg) at Week 72, compared to 35.2% on placebo [22, 23]. Mediation analysis in SURMOUNT‐1 attributed about 68% (SBP) and 71% (DBP) of these improvements to weight loss, highlighting the practical benefit of the drug on BP categories, not just mean values.

In a secondary analysis of SUMMIT, tirzepatide at 52 weeks significantly reduced SBP relative to placebo (estimated treatment difference ~−5 mmHg) and markedly reduced estimated circulating blood volume by ~0.58 L [24]. This suggests tirzepatide relieves volume overload and vascular stiffness in HFpEF, contributing to its BP‐lowering effect and symptomatic benefit.

In pooled analyses, tirzepatide‐induced BP declines did not cause symptomatic hypotension in most patients [20]. Nevertheless, clinicians should monitor for orthostatic symptoms when initiating tirzepatide in patients on multiple BP drugs (especially diuretics or SGLT2 inhibitors) and adjust co‐medications if needed. Also, small dose‐dependent increases in heart rate have been observed in trials, with no consistent increase in atrial fibrillation in meta‐analyses [25, 26].

Taken together, BP lowering plus improvements in weight and lipids likely reduce HF and stroke risk and improve HFpEF symptoms, with the greatest benefit in patients starting with higher SBP and obesity‐related haemodynamic load [19, 20, 24].

### 3.2 Tirzepatide in Weight and Body Composition

In overweight or obese patients without diabetes (SURMOUNT‐1), once‐weekly tirzepatide (up to 15 mg) induced dose‐dependent and sustained weight loss over 72 weeks: −15.0% (5 mg), −19.5% (10 mg), −20.9% (15 mg) versus −3.1% with placebo. Notably, ≥ 20% loss was achieved in 50%–57% of patients at higher doses. This profound weight loss unloads the circulation, reduces visceral and cardiac fat, and improves ventricular filling pressures—all central to HFpEF pathophysiology.

In SUMMIT, tirzepatide resulted in (−13.9% vs. −2.2% in placebo) mean weight loss at 52 weeks, with corresponding improvements in symptoms and a lower rate of death from cardiovascular causes or worsening heart‐failure events (9.9% vs. 15.3% in placebo) [27]. Consistent with these clinical benefits, the SUMMIT CMR substudy found that tirzepatide significantly reduced left ventricular (LV) mass (~−11 g) and paracardiac (epicardial plus pericardial) adipose volume (~−45 mL) compared to placebo [28]. The LV mass change correlated with weight loss, supporting a weight‐mediated reverse remodelling effect.

### 3.3 Tirzepatide in Lipids and Atherogenic Particles

Across SURPASS trials, tirzepatide treatment produced significant improvements in atherogenic lipids: total cholesterol fell (TC −3.8%/−4.6%/−5.9% at 5/10/15 mg) with reduced triglycerides and LDL‐C. HDL‐C was increased [19]. These lipid improvements are largely weight‐loss–mediated, as noted in SURPASS‐J.

In a post hoc analysis of SURPASS J‐mono, greater weight loss on tirzepatide was associated with larger improvements in triglycerides and HDL‐C [21]. By lowering triglyceride‐rich lipoproteins (a source of residual risk), tirzepatide can complement LDL‐cholesterol–targeted therapy to slow atherosclerotic progression.

In another post hoc analysis of the phase‐2 tirzepatide study, tirzepatide dose‐dependently reduced apoC‐III and apoB and lowered the number of large triglyceride‐rich lipoprotein and small LDL particles, consistent with decreased remnant cholesterol and an overall less atherogenic profile [29].

### 3.4 Tirzepatide in Cardiovascular Outcomes

Complementary evidence comes from SURPASS‐CVOT, an event‐driven cardiovascular outcomes trial in > 13,000 patients with type 2 diabetes and established atherosclerotic cardiovascular disease, which compared tirzepatide (up to 15 mg weekly) with dulaglutide 1.5 mg weekly, a GLP‐1 receptor agonist with proven CV benefit [9]. The design and baseline characteristics have been published, confirming a high‐risk cohort with long‐standing diabetes and established ASCVD. According to the first peer‐reviewed report of the top‐line results, tirzepatide met the primary endpoint of non‐inferiority for 3‐point MACE (CV death, nonfatal myocardial infarction or nonfatal stroke) compared with dulaglutide, with an approximately 8% relative risk reduction (hazard ratio ~0.92; 95% CI 0.83–1.01) [30]. Thus, tirzepatide preserved at least the full cardioprotective effect of a benchmark GLP‐1 RA, with no signal of increased cardiovascular risk. Exploratory analyses reported numerically lower all‐cause mortality, larger reductions in HbA1c and body weight, and signals for slower decline in estimated glomerular filtration rate in high‐risk subgroups with tirzepatide versus dulaglutide, consistent with its broader metabolic effects.

### 3.5 Tirzepatide in Renal Function and Outcomes

Tirzepatide was associated with a significantly greater reduction in the urine albumin‐to‐creatinine ratio (UACR) compared with controls, with an average decrease of approximately 26.9%. Among participants with baseline UACR levels ≥ 30 mg/g, the reduction was even more pronounced at around 41.4% [31].

In a post hoc analysis of the SURPASS‐4 trial, which included patients with type 2 diabetes, a body mass index (BMI) ≥ 25 kg/m^2^, and high cardiovascular risk, once‐weekly tirzepatide (5, 10 or 15 mg) was compared with insulin glargine. Over a median follow‐up of 85–104 weeks, tirzepatide treatment was associated with a lower incidence of the composite kidney endpoint, defined as an eGFR decline of ≥ 40%, renal death, progression to kidney failure or new‐onset macroalbuminuria [32].

**FIGURE 3:**
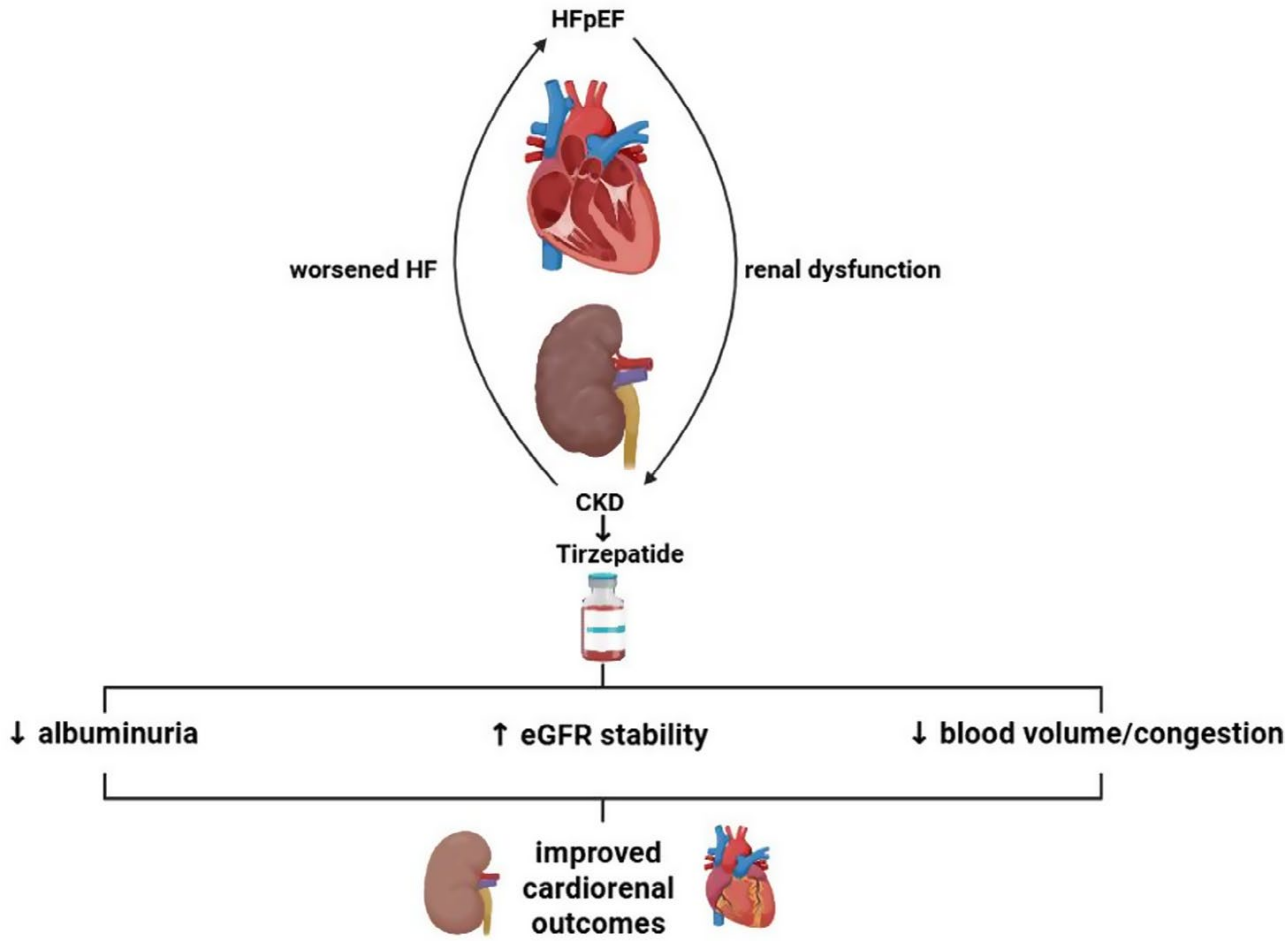
Cardiorenal benefits of tirzepatide. By reducing volume overload, albuminuria and stabilising kidney function, tirzepatide addresses the bidirectional relationship between heart failure with preserved ejection fraction and chronic kidney disease.

Nearly 60% of enrolled patients had chronic kidney disease, and in this subgroup tirzepatide conferred similar reductions in HF events compared with patients without renal impairment. A dedicated renal analysis showed that after an initial dip in eGFR, consistent with haemodynamic adjustment, tirzepatide stabilised or improved kidney function over 52 weeks relative to placebo. A reduction in albuminuria was also observed, suggesting potential renal protective effects alongside the cardiac benefits [33] (Figure 3).

### 3.6 Tirzepatide in Inflammation, Endothelial Function and Vascular Remodelling

Obesity is characterised by chronic low‐grade inflammation and oxidative stress that worsen atherosclerosis and HFpEF. Across randomised human studies, tirzepatide consistently attenuates systemic inflammation (Table 1).

**TABLE 1:** Anti‐inflammatory and anti‐oxidative effects of tirzepatide.

Trial/Population	72‐week IL‐6 change vs. placebo	72‐week hsCRP change vs. placebo	Mediation by weight loss
SURMOUNT‐1 (obesity, no T2D)	↓ 26%–31%	↓ 51%–65%	24 weeks: ~18% • 72 weeks: IL‐6 77%, hsCRP 87%
SURMOUNT‐2 (T2D)	↓ 16%–23% (15 mg IL‐6 NS)	↓ 55%–56%	24 weeks: ~31% • 72 weeks: IL‐6 78%, hsCRP 57%

Abbreviations: IL, interleukin; T2D, type 2 diabetes mellitus.

In SURMOUNT, 72‐week changes versus placebo were substantial: IL‐6 ↓ ~ 26%–31% and hsCRP ↓ ~51%–65%. Mediation analyses show that early (24‐week) declines are only modestly weight‐mediated (≈18%–31%), whereas by 72 weeks the reductions are largely weight‐mediated (≈57%–87%), indicating an initial partly weight‐independent signal that becomes predominantly weight‐linked with sustained treatment [34].

In a phase‐2 randomised programme, tirzepatide also lowered ICAM‐1 and YKL‐40 dose‐dependently by 26 weeks, alongside hsCRP reductions. Thus, supporting broader immunometabolic modulation beyond CRP/IL‐6 [29].

In SUMMIT, tirzepatide reduced hsCRP by ~37% and high‐sensitivity troponin‐T by ~10% and lowered systolic blood pressure by ~5 mmHg versus placebo, while NT‐proBNP showed a modest/non‐significant decline (*p* ≈ 0.07). Importantly, ΔCRP correlated with both improved 6‐min walk distance and lower troponin, linking reduced systemic inflammation to less myocardial injury and better functional status [24].

As context, the GLP‐1RA class shows a compatible human signal; in a randomised trial, exenatide reduced hsCRP and MCP‐1 and lowered 8‐iso‐PGF2α (oxidative stress) over 16 weeks versus placebo, and meta‐analyses confirm significant CRP reductions with GLP‐1Ras; reinforcing a class anti‐inflammatory effect relevant to tirzepatide [35, 36].

Endothelial dysfunction is central to vascular stiffness and impaired ventricular–arterial coupling in HFpEF. Across randomised trials, GLP‐1 receptor agonists improve endothelial‐dependent vasodilation and, in aggregate, show modest improvements in arterial stiffness.

A 2024 network meta‐analysis of 38 RCTs in type 2 diabetes reported significant gains in brachial flow‐mediated dilation (FMD) and reductions in pulse‐wave velocity (PWV) versus placebo, consistent with enhanced NO‐dependent vasodilation [37]. In a 12‐week randomised study in type 1 diabetes, once‐weekly semaglutide nearly doubled FMD (≈5.8% → ≈11.1%; *p* < 0.001) and lowered peripheral vascular resistance (~5%; *p* ≈ 0.046), demonstrating measurable endothelial benefit over a short horizon [38]. However, a separate 2024 meta‐analysis found no significant pooled effect of GLP‐1RAs on PWV, highlighting heterogeneity across populations, follow‐up duration and measurement methods [39].

For tirzepatide specifically, dedicated human FMD/PWV records are not yet available, but multiple signals point towards favourable vascular biology. In a phase‐2 randomised programme, tirzepatide reduced ICAM‐1 (with no change in VCAM‐1) alongside hsCRP reductions by 26 weeks. These findings are compatible with lower endothelial inflammatory activation [29].

Taken together, class effects on FMD/PWV plus tirzepatide's endothelial biomarker and haemodynamic profile support a model in which tirzepatide enhances vascular compliance indirectly (via weight loss, lower BP and reduced inflammation) and likely directly through endothelial pathways, thereby helping to reduce afterload and support diastolic filling pressures in HFpEF.

## 4 Implications for Heart Failure Management

**FIGURE 4:**
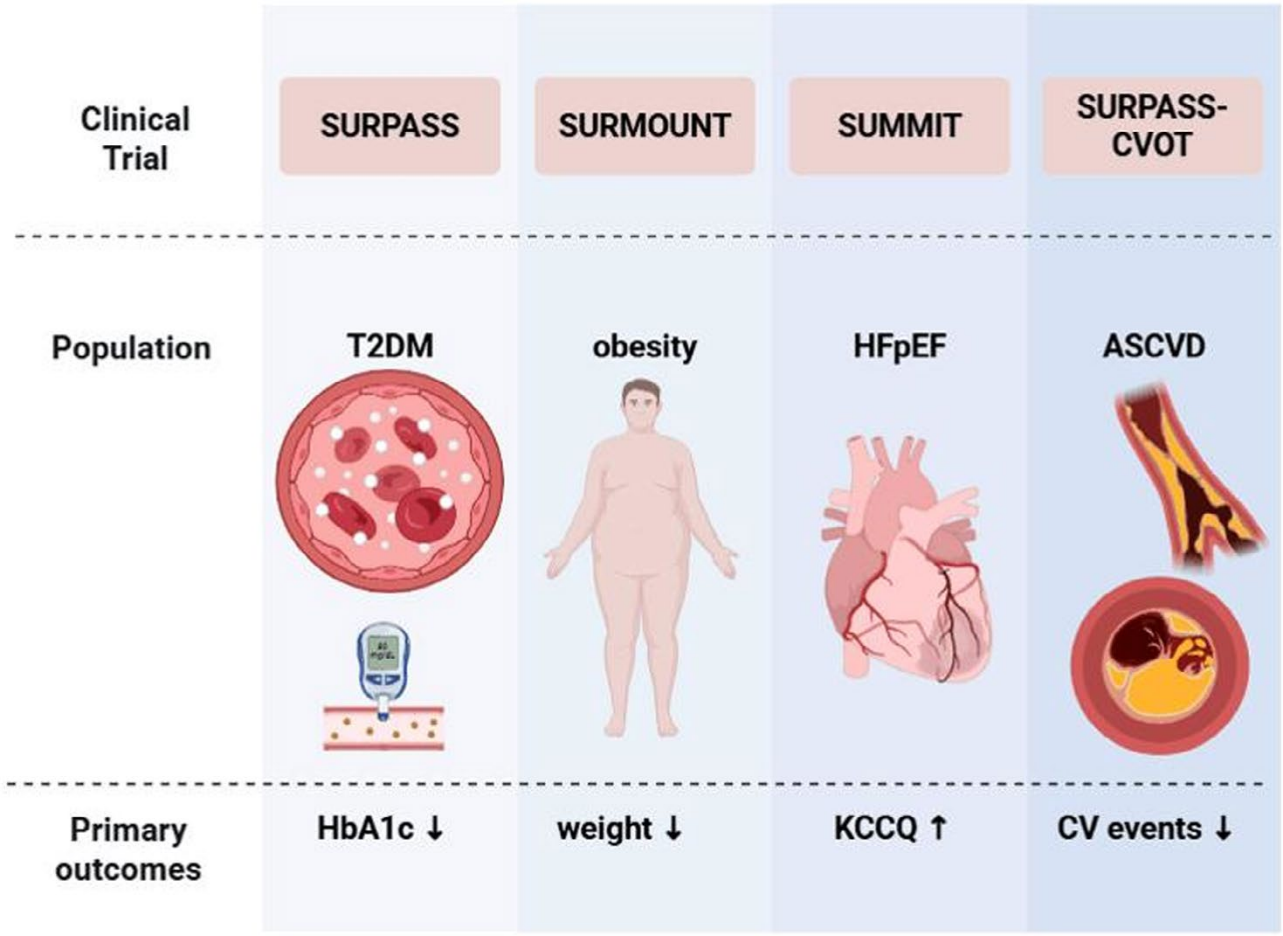
Overview of key tirzepatide clinical trials and their cardiovascular and metabolic outcomes. The SURPASS programme (T2DM), SURMOUNT (obesity), SUMMIT (HFpEF) and SURPASS‐CVOT (ASCVD) consistently demonstrate benefits on glycemic control, weight reduction, cardiovascular outcomes and renal function.

Addressing obesity and metabolic dysfunction has become a key therapeutic focus in HF, as evidence is mounting that treating these metabolic drivers can improve HF outcomes. Tirzepatide, through its potent weight loss and metabolic benefits, offers a novel therapeutic approach that is particularly relevant to HF with preserved ejection fraction (HFpEF) (Figure 4, Table 2).

**TABLE 2:** summary of recent evidence of tirzepatide in heart failure.

Title	Design	Sample size	Setting	Study period	Eligibility	Intervention/Exposure	Comparator	Primary outcomes	Secondary outcomes	Follow‐up window	Main results	Takeaway conclusion
Real‐world efficacy of tirzepatide in patients with heart failure without diabetes	Retrospective cohort	Before matching: Non‐tirzepatide 471,830 vs. Tirzepatide 904 After matching: 897 vs. 897 (*n* = 1794)	USA	Jan 1, 2013–Dec 1, 2024	Adults 18–70 with HF; excluded diabetes (diagnosis, HbA1c > 6.5%, diabetes meds); excluded other GLP‐1 users	Prescription of tirzepatide (real‐world, dose details not available)	Patients with HF and no tirzepatide prescription (propensity matched)	Incident acute heart failure events	MACE, CKD progression, stroke, CAD, incident T2DM, all‐cause mortality, PAD	Up to 4 years after index	Tirzepatide associated with lower incidence of acute HF vs. non‐users (non‐user vs. user HR 3.12, 95% CI 2.24–4.35) Lower major adverse cardiovascular events (MACE) in tirzepatide users (non‐user vs. user HR 3.57, 95% CI 2.32–5.48) Reduced stroke risk in tirzepatide group (non‐user vs. user HR ≈ 2.8) Fewer CKD progression and CAD events observed with tirzepatide (HRs ~1.48 and ~1.47 for untreated vs. treated)	Tirzepatide use in HF patients without diabetes was associated with markedly lower acute HF and MACE vs. matched non‐users; findings require prospective randomised confirmation
Effects of tirzepatide on circulatory overload and end‐organ damage in HFpEF with obesity: secondary analysis of SUMMIT	Secondary analysis of randomised, double‐blind, placebo‐controlled SUMMIT trial	731 randomised (Tirzepatide *n* = 364; Placebo *n* = 367)	129 centres across nine countries (multicentre, international)	Enrolment Apr 20, 2021–Jun 30, 2023; mechanistic endpoints to 52 weeks	Age ≥ 40, HF NYHA II–IV, HFpEF (LVEF ≥ 50%), BMI ≥ 30 kg/m^2^, 6MWD 100–425 m, KCCQ‐CSS ≤ 80 Objective HF evidence (elevated NT‐proBNP, LA enlargement, or elevated filling pressures) Enrichment if recent HF decompensation or eGFR < 70	Tirzepatide SC weekly (dose titrated from 2.5 mg → up to 15 mg weekly [increase 2.5 mg every 4 weeks as tolerated]) + usual care	Matching placebo + usual care	CV death or worsening HF and KCCQ change (mechanistic endpoints—BP, estimated BV/PV, hsCRP, troponin, NT‐proBNP, eGFR, UACR, 6MWD, KCCQ)	Biomarker changes, renal indices, exercise capacity, QoL and correlations among changes	Mechanistic assessments at 12, 24, 52 weeks; events followed throughout trial	Systolic BP reduced with tirzepatide (ETD −5 mmHg at 52 weeks)Estimated blood volume decreased (ETD −0.58 L at 52 weeks) Systemic inflammation fell (hsCRP −37% at 52 weeks) Myocardial injury marker troponin T decreased (−10.4% at 52 weeks); NT‐proBNP trended down (−10.5%, *P* ≈ 0.07) Renal indices improved: eGFR +2.9 mL min^−1^ 1.73 m^−2^ at 52 weeks; UACR −25% at 24 weeks and −15% at 52 weeks Reductions in estimated blood volume correlated with improved KCCQ and 6MWD	In obese HFpEF, tirzepatide reduced circulatory volume, systemic inflammation, and markers of myocardial/renal injury with correlated symptomatic and functional improvements—supporting mechanisms for reduced worsening HF
Interplay of chronic kidney disease and the effects of tirzepatide in HFpEF and obesity (SUMMIT subgroup analysis)	Pre‐specified/post‐hoc subgroup analyses of the randomised SUMMIT RCT	731 randomised (Tirzepatide *n* = 364; Placebo *n* = 367)	129 centres across nine countries (multicentre, international)	Randomisation Apr 20, 2021–Jun 30, 2023; biomarker/clinical follow‐up to 52 weeks (some outcomes to ~104 weeks)	Age ≥ 40, HF NYHA II–IV, HFpEF (LVEF ≥ 50%), BMI ≥ 30 kg/m^2^, 6MWD 100–425 m, KCCQ‐CSS ≤ 80 Objective HF evidence (elevated NT‐proBNP, LA enlargement, or elevated filling pressures) Enrichment if recent HF decompensation or eGFR < 70	Tirzepatide SC weekly (dose titrated from 2.5 mg → up to 15 mg weekly [increase 2.5 mg every 4 weeks as tolerated]) + usual care	Matching placebo + usual care	Influence of CKD status on CV death/worsening HF and modification of tirzepatide effects; serial changes in renal function (eGFR‐creatinine/cystatin C) and UACR	KCCQ, 6MWD, hsCRP, safety signals by CKD strata	Clinical follow‐up through trial duration (median ~104 weeks for some analyses); biomarkers at 12, 24, 52 weeks	CKD prevalence higher when estimated by cystatin C vs. creatinine (61% vs. 46%) Patients with CKD had worse baseline function and ~2× higher risk of worsening HF Relative benefit of tirzepatide on CV death/worsening HF preserved across CKD strata; absolute risk reduction larger in CKD patients Transient dip in eGFR‐creatinine at 12 weeks, but eGFR improved by 52 weeks (especially by cystatin C) Discordance between creatinine and cystatin C suggests body‐composition changes (weight/muscle loss) affect creatinine‐based eGFR; interpret renal changes cautiously during weight loss	Tirzepatide benefits for HF outcomes are maintained in patients with CKD and may yield renal function improvement long‐term, but eGFR interpretation during weight‐loss requires caution
Tirzepatide for Heart Failure with Preserved Ejection Fraction and Obesity	International, randomised, double‐blind, placebo‐controlled trial	731 randomised (Tirzepatide *n* = 364; Placebo *n* = 367)	129 centres across nine countries (multicentre, international)	Enrolment Apr 20, 2021–Jun 30, 2023; median follow‐up ≈104 weeks	Age ≥ 40, HF NYHA II–IV, HFpEF (LVEF ≥ 50%), BMI ≥ 30 kg/m^2^, 6MWD 100–425 m, KCCQ‐CSS ≤ 80 Objective HF evidence (elevated NT‐proBNP, LA enlargement or elevated filling pressures) Enrichment if recent HF decompensation or eGFR < 70	Tirzepatide SC weekly (dose titrated from 2.5 mg → up to 15 mg weekly [increase 2.5 mg every 4 weeks as tolerated]) + usual care	Matching placebo + usual care	Time to first adjudicated CV death or worsening HF event (hospitalisation/IV therapy/diuretic intensification) Change in KCCQ‐CSS from baseline to 52 weeks	6‐min walk distance at 52 weeks, percent change in body weight at 52 weeks, percent change in hs‐CRP at 52 weeks	Median follow‐up: 104 weeks	Composite (CV death or worsening HF): 9.9% tirzepatide vs. 15.3% placebo; HR 0.62 (95% CI 0.41–0.95), *p* = 0.026 Worsening HF events: 8.0% vs. 14.2%; HR 0.54 (95% CI 0.34–0.85) Cardiovascular deaths: 8 vs. 5 (imprecise; HR 1.58, 95% CI 0.52–4.83) KCCQ‐CSS at 52 weeks: mean change + 19.5 (tirzepatide) vs. + 12.7 (placebo); between‐group +6.9 points (95% CI 3.3–10.6), *p* < 0.001 Body weight (52 weeks): −13.9% vs. −2.2%; between‐group ≈ −11.6 percentage points, *p* < 0.001 hs‐CRP (52 weeks): −38.8% vs. −5.9% (between‐group −34.9 pp), *p* < 0.001 Safety: more discontinuations for adverse events (mainly GI) with tirzepatide (6.3% vs. 1.4%); all‐cause deaths 19 vs. 15 (HR 1.25, 95% CI 0.63–2.45)	In HFpEF patients with obesity, weekly tirzepatide (up to 15 mg) reduced the risk of CV death/worsening HF and produced clinically meaningful improvements in symptoms, exercise capacity, weight, and inflammation at 52 weeks; GI adverse effects increased discontinuations. Mortality benefit uncertain and applicability to lower‐BMI HFpEF is unknown
Effects of Tirzepatide on the Clinical Trajectory of Patients With Heart Failure, Preserved Ejection Fraction, and Obesity	Prespecified expanded analyses and sensitivity analyses of the SUMMIT randomised, double‐blind trial	731 randomised (Tirzepatide *n* = 364; Placebo *n* = 367)	129 centres across nini countries (multicentre, international)	Enrolment Apr 20, 2021–Jun 30, 2023	Age ≥ 40, HF NYHA II–IV, HFpEF (LVEF ≥ 50%), BMI ≥ 30 kg/m^2^, 6MWD 100–425 m, KCCQ‐CSS ≤ 80 Objective HF evidence (elevated NT‐proBNP, LA enlargement, or elevated filling pressures) Enrichment if recent HF decompensation or eGFR < 70	Tirzepatide SC weekly (dose titrated from 2.5 mg → up to 15 mg weekly [increase 2.5 mg every 4 weeks as tolerated]) + usual care	Matching placebo + usual care	Contextualised to SUMMIT primaries (CV death/worsening HF and KCCQ‐CSS at 52 weeks); this paper emphasises expanded outcomes and sensitivity analyses	6MWD, EQ‐5D‐5L, PGIS Overall Health, NYHA class shifts, medication intensifications/changes, hierarchical composite/win‐ratio, NT‐proBNP	Median follow‐up: 104 weeks	KCCQ‐CSS (52 weeks): between‐group +6.9 points (95% CI 3.3–10.6; *p* < 0.001); on‐treatment estimate ≈ + 9.8. Larger proportion achieved ≥ 20‐point improvement (47.6% vs. 35.2%) 6MWD (52 weeks): median between‐group +18.3 m (95% CI 9.9–26.7), *p* < 0.001; more achieved ≥ 25 m improvement (51.7% vs. 34.0%) EQ‐5D‐5L and PGIS: meaningful improvements (EQ‐5D diff 0.06; PGIS OR 1.99). NYHA class shifts favoured tirzepatide (OR 2.26) Medication burden: fewer HF medication intensifications and fewer diuretic dose increases; more diuretic reductions in tirzepatide group (*p* = 0.015) NT‐proBNP: modest (~10%) decreases; borderline significance across time points	Expanded analyses demonstrate broad, consistent benefits of tirzepatide across events, symptoms, exercise tolerance, quality of life and reduced medication intensification in HFpEF patients with obesity—indicating a favourable shift in the clinical trajectory; longer‐term durability and applicability to non‐obese HFpEF need further study

### 4.1 Tirzepatide and HFpEF

The SUMMIT trial was the first large, double‐blind outcomes study of dual‐incretin therapy in obesity‐related HFpEF. In this study, just over 700 symptomatic patients with HFpEF (LVEF ≥ 50%) and BMI ≥ 30 kg/m^2^ were randomised to weekly tirzepatide or placebo and followed for a median of ~2 years.

Tirzepatide significantly reduced the prespecified composite of cardiovascular death or worsening heart‐failure events by 38% versus placebo—an effect driven predominantly by fewer HF hospitalizations and urgent HF visits, while all‐cause and cardiovascular mortality were neutral. Treatment was also associated with marked weight loss (≈12%–14% at 52 weeks) and clinically meaningful improvements in symptoms and exercise capacity. The magnitude of weight reduction approaches that seen after bariatric surgery in some series [1, 40].

Mechanistic analyses from SUMMIT provide insight into how tirzepatide achieves these benefits. The drug significantly reduced estimated blood volume, lowered high‐sensitivity troponin levels and attenuated systemic inflammation. These changes are consistent with relieving volume‐pressure overload and myocardial stress [24].

**FIGURE 5:**
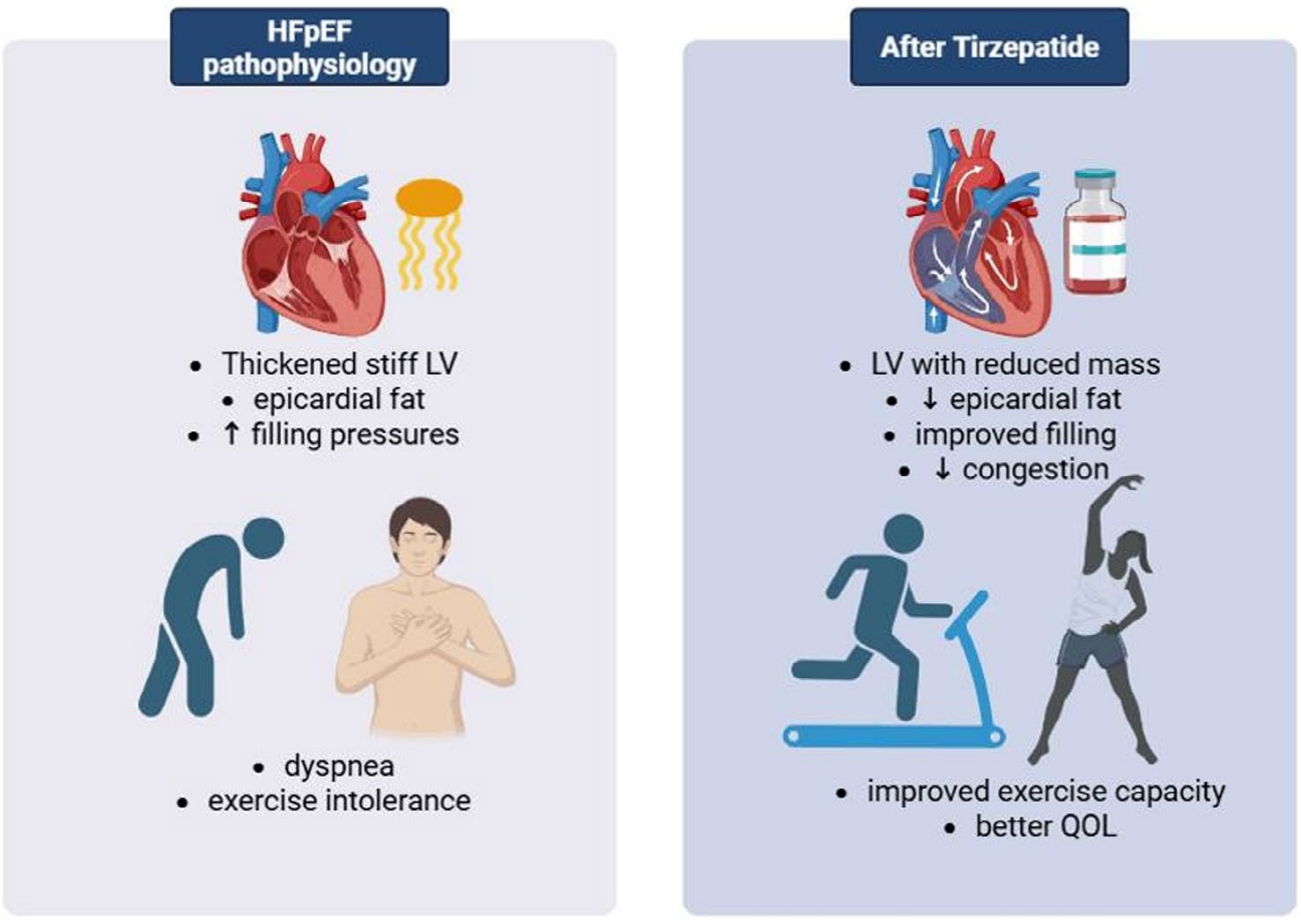
Schematic illustration of Tirzepatide's effects on heart failure with preserved ejection fraction (HFpEF). By inducing weight loss, reducing epicardial adiposity, lowering blood volume and reversing left ventricular remodelling, tirzepatide improves diastolic function, reduces congestion and enhances functional outcomes.

An expanded secondary analysis confirmed that tirzepatide's benefits extended across multiple domains of clinical status, including improved quality of life, functional class and a reduced need for HF medications [41]. Furthermore, a cardiac MRI substudy demonstrated reverse remodelling: tirzepatide therapy led to regression of left ventricular mass and a reduction in paracardiac adipose tissue (LV mass −11 g; paracardiac fat −45 mL) [28]. Such structural improvements likely translate into better diastolic function and symptomatic relief (Figure 5).

Collectively, SUMMIT offers high‐quality evidence that targeting obesity and metabolic dysfunction with a dual GIP/GLP‐1 agonist can modify the disease trajectory in HFpEF, reducing HF events while improving exercise capacity and quality of life.

Complementary real‐world evidence comes from a brief report by Kishimori et al. [42], who compared tirzepatide with semaglutide in adults with HFpEF and type 2 diabetes using the global TriNetX electronic health record network. After propensity score matching (*n* ≈ 5800 per group), 1‐year risks of acute heart failure, all‐cause death and all‐cause hospitalisation did not differ significantly between tirzepatide and semaglutide (HR for acute HF 0.98, 95% CI 0.89–1.07; HR for all‐cause death 0.87, 95% CI 0.67–1.13; HR for all‐cause hospitalisation 1.00, 95% CI 0.94–1.06), and gastrointestinal adverse events and hypoglycaemia were also similar. These findings suggest that in HFpEF with diabetes, tirzepatide and semaglutide provide broadly comparable short‐term HF and survival outcomes, reinforcing a class cardiometabolic benefit while allowing weight‐loss and metabolic responses to guide individual drug selection.

Recognising this evidence, new guidelines have begun to integrate obesity management into HF care. The 2025 ACC scientific statement on obesity in heart failure addresses the therapeutic role of GLP‐1 and dual GIP/GLP‐1 agonists for patients with HFpEF and obesity, and it recommends multidisciplinary care pathways to integrate anti‐obesity medications safely and effectively [43].

For clinicians managing patients with HF, who frequently have concomitant obesity, diabetes and ischemic heart disease, these data suggest that using tirzepatide for weight and glycemic control is unlikely to compromise cardiovascular outcomes and may provide incremental reductions in adverse events on top of standard HF therapies [9, 30]. When viewed alongside HFpEF trials such as SUMMIT and STEP‐HFpEF, the emerging picture is that dual incretin agonism can simultaneously improve HF‐related symptoms and haemodynamics in obesity‐related HFpEF while lowering long‐term atherosclerotic event risk without an apparent trade‐off in cardiovascular safety.

### 4.2 Implications in HFrEF

In contrast to the HFpEF findings, the role of incretin‐based therapies in heart failure with reduced ejection fraction (HFrEF) remains uncertain. Earlier trials of GLP‐1 receptor agonists in HFrEF, most notably FIGHT, showed no clinical benefit [44]. Also, the LIVE trial reported higher resting heart rate and more serious cardiac events with liraglutide versus placebo [45], supporting a cautious approach in this phenotype.

In FIGHT, liraglutide did not improve outcomes and there were signals of possible harm, such as a higher incidence of HF hospitalisation [44]. In addition, liraglutide did not improve left ventricular ejection fraction and was associated with an ≈7 bpm increase in resting heart rate and more serious cardiac adverse events compared with placebo [45]. To date, no large randomised outcomes trial of tirzepatide has been performed specifically in HFrEF, and patients with severely reduced EF or recent decompensation were generally excluded from tirzepatide's studies.

An ‘obesity paradox’ has been noted in HFrEF; higher body mass often correlates with better outcomes, so rapid weight loss could unmask frailty or cachexia in this population [46, 47]. Until dedicated HFrEF trials are conducted, any tirzepatide use in such patients should be very cautious and highly individualised. Consistently, a recent meta‐analysis of randomised trials in HFrEF found no improvement in key HF outcomes with GLP‐1 receptor agonists [48].

Emerging real‐world data suggest that tirzepatide may be safe, and possibly beneficial, even in patients with advanced systolic heart failure and ventricular arrhythmias, with a propensity score–matched analysis reporting lower 1‐year mortality in tirzepatide users compared with non‐users. However, these observational, arrhythmia‐focused cohorts are prone to confounding and do not overturn the neutral or negative randomised trial data; they are better interpreted as reassuring for cardiovascular safety when tirzepatide is used to treat coexisting obesity or diabetes in HFrEF rather than as evidence of HF‐specific efficacy [49].

For HFrEF patients with severe obesity, tirzepatide may still be considered on a case‐by‐case basis when excess adiposity is a clear driver of haemodynamic compromise or refractory symptoms. In such cases, a collaborative approach (involving cardiology, endocrinology, nutrition, etc.) is advised, with close monitoring and dose adjustments as weight and haemodynamic changes.

### 4.3 Impact on Quality of Life and Functional Capacity

In HFpEF, where symptom burden and impaired functional capacity are often the main clinical concerns, the improvements observed with incretin therapy are particularly important. In SUMMIT, patients receiving tirzepatide reported substantially better health‐related quality of life, with Kansas City Cardiomyopathy Questionnaire (KCCQ) scores improving by nearly seven points more than placebo at 1 year, a change that exceeds the established threshold for clinical significance. This along with an average increase of ~18 m in the 6‐min walk test, a functional improvement rarely achieved with existing pharmacologic therapies in HfpEF [27]. Of note, the trajectory of benefit began within the first few months of treatment and was maintained throughout follow‐up, demonstrating both the efficacy and durability of this approach.

STEP‐HFpEF provides converging evidence with semaglutide, where patients also experienced clinically significant improvements in KCCQ scores and exercise capacity over 52 weeks. These benefits occurred alongside reductions in systemic inflammation (as reflected by high‐sensitivity CRP) and body weight, suggesting that the observed improvements in patient‐reported outcomes reflect both haemodynamic and systemic metabolic relief. Subgroup analyses further revealed that the magnitude of quality‐of‐life benefit correlated with baseline NT‐proBNP levels, indicating that patients with greater haemodynamic burden may derive disproportionate symptomatic improvement from incretin therapy [27, 40].

Trial readouts collectively support a pathway where substantial, sustained weight loss (plus anti‐inflammatory and vascular effects) improves filling pressures, exercise tolerance and daily function. SUMMIT's imaging substudy adds structure–function specificity (↓LV mass, ↓paracardiac fat), while STEP's biomarker/CRP data show important systemic anti‐inflammatory contribution. These mechanisms extended to cardiorenal syndrome indicated previously, where reduced venous congestion and improved metabolic status stabilise kidney function, consistent with SUMMIT's 52‐week eGFR findings [24, 33].

The signal hierarchy now spans (a) patient experience (KCCQ) and performance (6MWD) in STEP‐HFpEF, and (b) hard clinical events (HF worsening) plus patient‐reported outcomes in SUMMIT—together informing 2024–2025 scientific statements that place GLP‐1/dual GIP‐GLP‐1 agonists within multidisciplinary obesity management for HFpEF. Pending broader uptake with background SGLT2/MRA therapy and longer follow‐up for mortality, the consistent quality‐of‐life and functional gains justify considering incretin therapy when excess adiposity is the driver of limitation.

## 5 Comparative Overview With Other GLP‐1 Agents: Tirzepatide Versus Semaglutide

### 5.1 Physiologic and Mechanistic Standpoint

Tirzepatide and semaglutide share a common GLP‐1–based backbone but differ in receptor profile and, potentially, in how they modify the heart–adipose–metabolic axis relevant to HF. Semaglutide is a selective GLP‐1 receptor agonist that promotes glucose‐dependent insulin secretion, suppresses glucagon, slows gastric emptying and reduces appetite, leading to substantial weight loss and improvements in glycemic control, blood pressure and systemic inflammation [40, 50]. GLP‐1 signalling also appears to exert natriuretic, endothelial and modest direct myocardial effects that may be particularly relevant in cardiometabolic HFpEF [13, 14].

Tirzepatide is a dual GIP/GLP‐1 receptor agonist. In addition to GLP‐1–mediated effects, agonism at the GIP receptor further enhances insulin secretion, improves insulin sensitivity and amplifies weight loss beyond that typically achieved with GLP‐1 monotherapy [13, 14, 51]. This more potent metabolic effect translates into larger reductions in body weight, visceral and ectopic fat, including pericardial/epicardial adipose tissue and left‐ventricular mass, as shown in the SUMMIT CMR substudy [52]. By aggressively unloading adiposity and improving cardiometabolic risk factors (glycemia, blood pressure, inflammatory markers), tirzepatide and semaglutide both target the core pathophysiologic substrate of obesity‐related HFpEF rather than a narrow LVEF phenotype [40, 50, 53, 54].

### 5.2 Trial Data and Clinical Outcomes

Both tirzepatide and semaglutide have robust randomised evidence in obesity‐related HFpEF. In STEP‐HFpEF, semaglutide 2.4 mg weekly produced clinically meaningful improvements in health status and function (placebo‐corrected KCCQ‐CSS ≈ +7–8 points; 6MWD ≈ +15–20 m), ≈13% mean weight loss versus ≈2%–3% with placebo and larger reductions in NT‐proBNP (~20% vs. ~5%) [40, 50, 53, 54].

In SUMMIT (*n* = 731, LVEF ≥ 50%), tirzepatide (up to 15 mg weekly) lowered the composite of cardiovascular death or worsening HF from 15.3% to 9.9% (HR 0.62; 95% CI 0.41–0.95), driven by fewer HF hospitalizations/urgent visits; it also yielded greater KCCQ gains (~19.5 vs. 12.7 points), modest 6MWD increases (~15–20 m), ≈12%–15% weight loss, and CMR‐documented reductions in LV mass and paracardiac fat [27, 52].

In SURMOUNT‐5, a phase 3b, open‐label trial in adults with obesity (mean BMI ~39.5 kg/m^2^) without established cardiovascular disease or type 2 diabetes, tirzepatide was compared head‐to‐head with semaglutide for weight loss and modelled 10‐year cardiovascular risk reduction [55]. In a post hoc analysis, presented at ESC 2025 and including ~750 participants who completed 72 weeks of treatment, 10‐year cardiovascular disease (CVD) risk was estimated using validated BMI‐based Framingham equations. Baseline predicted risk was approximately 9%. Tirzepatide produced a larger absolute reduction in 10‐year CVD risk than semaglutide (about −2.4% vs. −1.4%; *p* < 0.001), corresponding to a relative risk reduction of roughly 24% versus ~14% with semaglutide. These differences were largely driven by greater weight loss and cardiometabolic improvements (blood pressure, glycemia, lipids) in the tirzepatide arm, and population‐level modelling suggested that, in an eligible U.S. population, tirzepatide could potentially prevent nearly twice as many CVD events over a decade as semaglutide. Although these estimates are based on risk modelling rather than adjudicated events, they illustrate how more intensive reductions in adiposity and risk factors may translate into larger long‐term cardioprotective gains, highly relevant given obesity's causal role in HFpEF and other cardiac phenotypes.

Overall, both agents produce similar placebo‐adjusted symptomatic and functional gains on a background of double‐digit weight loss; semaglutide offers more detailed NT‐proBNP data, while tirzepatide currently shows the clearest HF event‐reduction signal. Real‐world and emulation studies broadly corroborate lower HF and cardiorenal events with both drugs [40, 42, 51, 56].

### 5.3 Clinical Implications

From a clinical standpoint, tirzepatide and semaglutide can be viewed as disease‐modifying adjuncts in obesity‐driven HFpEF rather than ‘HF drugs’ in the traditional neurohormonal sense. Their main value lies in profound and sustained weight loss, improvement in metabolic risk factors, reduction of congestion and ectopic cardiac fat, and better functional capacity and health status [27, 40, 50, 52, 53].

In obese HFpEF with or without type 2 diabetes, either agent is reasonable in addition to guideline‐directed HF therapies (SGLT2 inhibitors, RAAS blockade, MRAs, diuretics). Tirzepatide may be favoured when very large weight loss or maximal glycemic improvement is a priority, or when the clinician wishes to leverage the HF event‐reduction signal seen in SUMMIT [27, 51, 52, 56]. Semaglutide may be preferred when a more extensive cardiovascular outcome portfolio is desired (e.g., SELECT and other CVOT data in ASCVD and obesity), or when natriuretic peptide reduction is particularly relevant [40, 50, 53, 57]. In HFrEF, use of either agent should remain individualised and metabolic‐driven (obesity, diabetes) rather than HF‐driven, pending dedicated outcome trials [44, 58].

## 6 Safety and Tolerability

**FIGURE 6:**
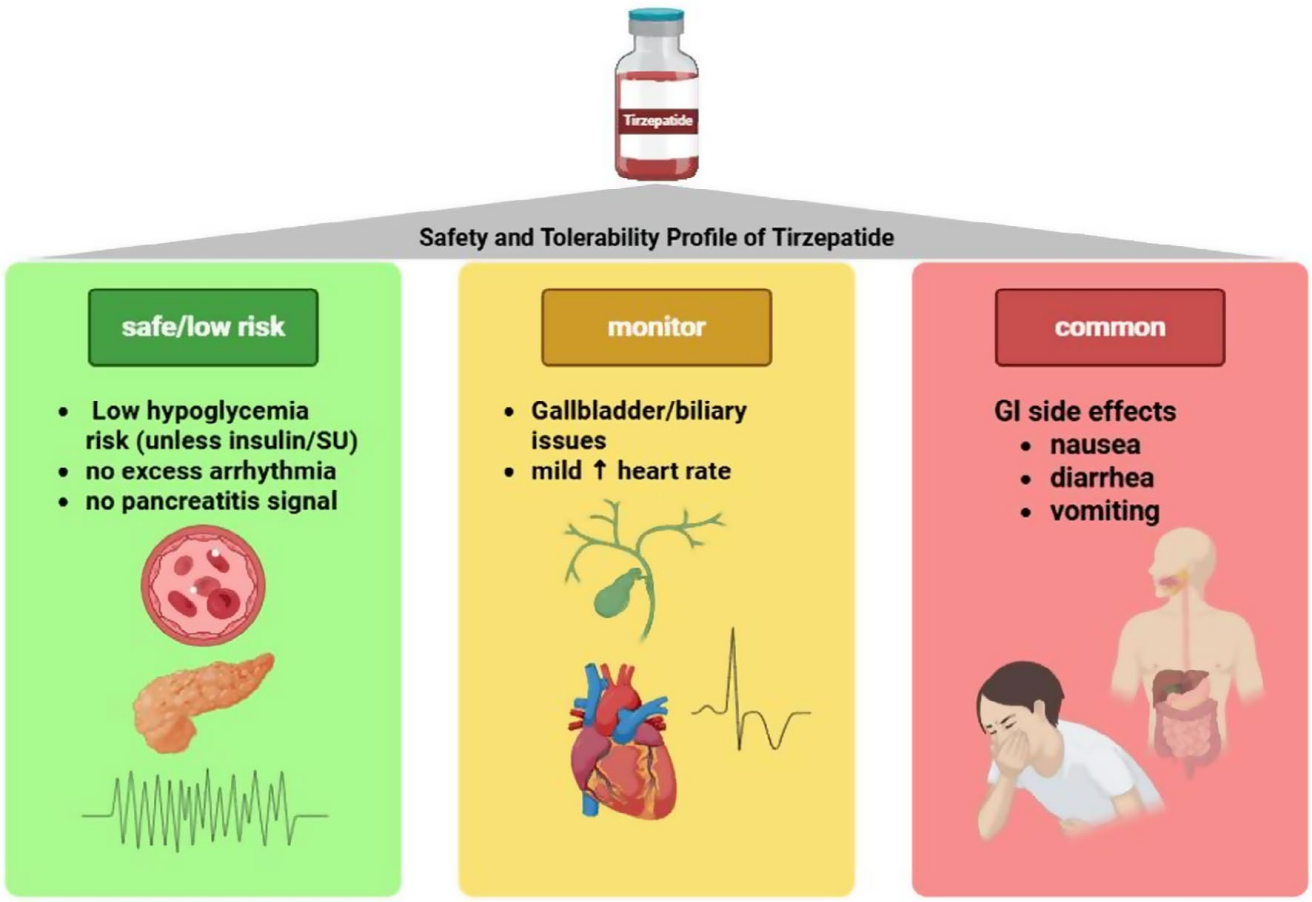
Safety and tolerability profile of tirzepatide. Gastrointestinal side effects are the most common but usually transient. Serious adverse events such as hypoglycaemia, pancreatitis, arrhythmia or biliary disease were not significantly increased compared to other therapies. Monitoring is recommended when combining with insulin, sulfonylureas or multiple antihypertensives.

The SURPASS clinical trial programmes examined the efficacy and safety of tirzepatide at three different doses (5, 10 and 15 mg) in T2DM patients. The recommended dose‐escalation method is to start with a dose of 2.5 mg weekly for the first 4 weeks followed by increments of 2.5 mg every 4 weeks until the maintenance dose based on the acceptable side effects [59]. Significant side effects were recorded by 1%–8% of participants with early or established diabetes and by 6%–17% of people with advanced diabetes during the SURPASS programme [60]. Gastrointestinal adverse events are the most commonly reported side effects of tirzepatide, including nausea, vomiting, diarrhoea, decreased appetite, constipation, dyspepsia and abdominal pain [61, 62]. These symptoms are dose dependent, and often attenuate with longer treatment duration, and some, such as nausea, diarrhoea and decreased appetite, may contribute to the weight‐reducing effects of tirzepatide [62]. At the 10 mg dose, tirzepatide may increase the risk of biliary events [63]. Neither cholelithiasis nor cholecystitis were associated significantly with tirzepatide use. In T2DM patients and obesity, there is no increased risk of pancreatitis. There has been no overall greater risk of biliary problems compared to insulin and GLP‐1 receptor agonists [63] (Figure 6).

Delayed gastric emptying increases the glucose‐dependent insulin secretion effect, results in patients being full earlier, which reduces the amount of food taken in; however, it can alter the absorption of orally administered drugs. This delay typically leads to diminished peak plasma concentration (*C*_max_) and prolongs time to reach peak levels (*T*_max_), whereas overall exposure (AUC) is usually not affected [64]. Clinically relevant effects have been mainly observed for orally administered drugs with a narrow therapeutic window, relying on rapid absorption, such as oral contraceptives, for which tirzepatide decreased both *C*_max_ and AUC. Oral contraceptives may not be adequately absorbed, which means pregnancy may not be prevented properly. Of particular note, manufacturer labelling for tirzepatide provides instructions that providers recommend the use of backup contraception in patients using oral hormonal contraceptives for 4 weeks after both drug initiation and dosage increase [65]. An approximately 20% reduction in overall exposure of oral contraceptives was reported following a single 5 mg dose of tirzepatide [66]. Therefore, careful consideration and possibly an alternative route of administration could be necessary when administering time‐ or narrow‐therapeutic‐index‐dependent medications together with tirzepatide. Furthermore, even though tirzepatide delays gastric emptying, which can increase residual gastric volume and theoretically increase aspiration risk during anaesthesia or deep sedation, current evidence does not show a statistically significant rise in actual aspiration events [67, 68].

In clinical trials of more than 4700 women, in which 3203 women received tirzepatide, only six pregnancies occurred, with one delivering a healthy baby, one spontaneously aborting, one ectopic pregnancy, one elective termination and two of unknown outcome, at rates comparable to placebo. However, because of the small number of pregnancies and lack of controlled human data, the potential foetal risk cannot be excluded [69]. Recommendations are to avoid tirzepatide during pregnancy, discontinuing it before conception unless the potential maternal benefit clearly outweighs the potential foetal risk, due to animal studies showing foetal growth restriction and developmental abnormalities at clinically relevant exposures.

The literature mentions that tirzepatide can cause mild to moderate hypoglycaemia as an adverse effect; however, the overall pattern suggests that it has a low risk of significant symptomatic hypoglycaemia, especially if not combined with insulin or sulfonylureas [70]. The incidence of serious hypoglycaemia in patients using tirzepatide was lower than that observed in patients using insulin glargine [60].

In addition, tirzepatide produced dose‐ and duration‐dependent thyroid C‐cell tumours in long‐term rat studies; human relevance is unknown but this finding underlies the warning and the contraindication in patients with a personal or family history of medullary thyroid carcinoma or MEN‐2. Routine calcitonin/ultrasound screening is of uncertain value; clinicians should counsel patients about symptoms of thyroid nodules and avoid use in those at risk [71]. Recent pooled analyses and systematic reviews did not report a consistent increase in thyroid cancer incidence in humans treated with tirzepatide over typical trial durations, but the evidence is imprecise and surveillance continues. Clinical trials including more than 4700 women (3203 of whom received tirzepatide) reported only six pregnancies occurring among tirzepatide users, which resulted in one healthy birth, one spontaneous abortion, one ectopic pregnancy, one elective termination and two with unknown outcomes, rates comparable to placebo. However, because of the limited number of pregnancies and no controlled human data, potential foetal risk cannot be excluded. Taken together with animal studies demonstrating foetal growth restriction and developmental abnormalities at clinically relevant exposures, current guidelines recommend avoiding tirzepatide in pregnancy and discontinuing it before conception unless the potential maternal benefit clearly outweighs the potential foetal risk [71].

Tirzepatide revealed overall cardiovascular safety in HFpEF patients [72, 73]. It did not increase cardiovascular mortality, there were no new ischemic events, and no signals of arrhythmogenicity were detected during patient follow‐up [72]. Even among HF patients, the leading cause of drug discontinuation was gastrointestinal side effects. Given the inadequate data on long‐term cardiovascular mortality, it is recommended that HF patients use the drug with caution [72].

Although the psychiatric adverse events are infrequent with tirzepatide, about 1%–2%, patients should nevertheless be monitored for depression or thoughts of suicide. Current evidence points towards low comparative risk relative to other GLP‐1 receptor agonists, but continued vigilance is nevertheless urged, particularly in those with prior psychiatric history [16, 74]. Additional studies in the future with extended follow‐up will be required to further evaluate the long‐term cardiovascular, renal, psychiatric and metabolic safety of tirzepatide and its safety in populations with low BMI HFpEF.

## 7 Future Directions and Unanswered Questions

The evidence reviewed in this narrative review confirms the potential of tirzepatide as a revolutionising treatment in HF, especially in patients with comorbidities such as T2DM and obesity. With the SUMMIT trial indicating significant improvement in outcomes among obese patients with HFpEF, forthcoming regulatory submissions to the FDA may render tirzepatide an approved therapy that specifically targets obesity‐associated HFpEF. Tirzepatide's effects on cardiovascular outcomes render it a valuable addition to current HF treatment algorithms. Although the benefits have been consistently proven, the exact mechanisms through which dual GIP/GLP‐1 receptor agonism provides cardiovascular protection are currently not well understood. Future mechanistic studies can provide insight into how tirzepatide fosters improvement in inflammation, cardiac remodelling and renal function. Data show tirzepatide achieves sustained weight loss, blood pressure, lipids, liver fat and kidney function improvements with decreased development of T2DM. Future research will be required to determine how these effects collectively contribute to reduction in long‐term risk of heart failure. Early data indicate tirzepatide has advantages over semaglutide in patients with HF and chronic kidney disease, with decreases in all‐cause mortality, stroke and myocardial infarction. Future trials are suggested to establish these potential differences. Recent evidence suggests that gastrointestinal side effects are usually temporary and resolve with time, while the risk for pancreatitis, gallbladder disease and hypoglycaemia appears comparable to GLP‐1 receptor agonists. Additional long‐term follow‐up is needed to validate the safety profile after 3 years of therapy.

## Author Contributions

Hamza A. Abdul‐Hafez and Ameer Awashra conceptualised the study, designed the review structure, supervised the writing process and critically revised the manuscript; Sosana Bdir, Sarah Saife, Qasem Salah, Mohammed Barbarawi and Thabet Swaileh participated in the literature search, data synthesis, writing and manuscript review; Ahmed Emara, Mohamed S. Elgendy and Abdalhakim Shubietah contributed to manuscript review, critical editing and interpretation of the findings. All authors contributed to the writing and review of the manuscript and approved the final version.

## Funding

The authors have nothing to report.

## Disclosure

Provenance and Peer Review: Not commissioned, externally peer‐reviewed.

## Ethics Statement

The authors have nothing to report.

## Consent

The authors have nothing to report.

## Conflicts of Interest

We affirm that this work is original, has not been published previously, and is not under consideration for publication elsewhere. None of the paper's contents have been previously published. All authors have read and approved the manuscript. We also provide full disclosure of any relationships with industry.

## Data Availability

The authors have nothing to report.
